# Effect of shunt surgery on idiopathic normal pressure hydrocephalus with incomplete Gerstmann syndrome: A CARE-compliant case report

**DOI:** 10.1097/MD.0000000000043669

**Published:** 2025-08-01

**Authors:** Kosei Goto, Nobuo Kutsuna, Takuto Nishihara, Kotaro Makita, Atsuya Suzuki

**Affiliations:** a Department of Neurosurgery, Fukujukai Adachi Tobu Hospital, Tokyo, Japan; b Department of Stress and Invasiveness Control, Toho University School of Medicine, Tokyo, Japan; c Department of Neurology, Umeda Clinic, Tokyo, Japan.

**Keywords:** case report, cognitive dysfunction, Gerstmann syndrome, idiopathic normal pressure hydrocephalus, shunt surgery

## Abstract

**Rationale:**

Idiopathic normal pressure hydrocephalus (iNPH) occasionally co-exists with neurodegenerative disease, but its concurrence with Gerstmann syndrome (GS) has not been reported, leaving the reversibility of GS-like deficits after cerebrospinal fluid diversion unknown.

**Patient concerns:**

A 77-year-old woman experienced a 1-year progressive decline in memory, object naming, and spatial orientation, eventually requiring institutional care.

**Diagnoses:**

Neurological examination revealed severe cognitive impairment (mini-mental state examination [MMSE] 4/30) with acalculia, agraphia, finger agnosia, and mild left–right disorientation, consistent with incomplete GS. Brain computed tomography demonstrated ventriculomegaly (Evans index 0.33), bilateral hippocampal atrophy, and a positive disproportionately enlarged subarachnoid space hydrocephalus sign, fulfilling Japanese iNPH criteria and suggesting comorbid Alzheimer’s disease.

**Interventions:**

In February 2024, a programmable ventriculoperitoneal shunt with a CODMAN CERTAS Plus valve was placed.

**Outcomes:**

At 1 month, MMSE improved to 9, and left–right disorientation, agraphia, and anomia resolved; finger agnosia recovered by 2 months, and acalculia partially improved by 3 months (MMSE 11). Serial imaging showed progressive resolution of the disproportionately enlarged subarachnoid space hydrocephalus sign, while ventricular size remained largely unchanged. No shunt-related complications occurred during the 3-month follow-up.

**Lessons:**

Ventriculoperitoneal shunting can reverse higher cortical dysfunction – including GS-like symptoms – in iNPH even when imaging suggests concomitant Alzheimer’s pathology. Recognition of such reversible neuropsychological signs may broaden surgical indications and improve patient outcomes.

## 1. Introduction

Idiopathic normal pressure hydrocephalus (iNPH) results in the expansion of the ventricles and typically presents with the classic triad of cognitive decline, gait disturbance, and urinary incontinence.^[1,2]^ Gait disturbance is often the initial symptom, manifesting as an unexplained symmetric gait impairment, which progresses and can become the most severe of the triad. Cognitive decline in iNPH tends to be mild compared with other dementias, resembling subcortical dementia, with memory impairment primarily characterized by recall difficulty, whereas recognition memory often remains intact.^[3,4]^ iNPH commonly occurs in older individuals,^[5,6]^ and shunt surgery is considered an effective treatment, with symptom improvement reported in 60% to 80% of patients. Hence, iNPH is recognized as a potentially reversible form of dementia.^[7,8]^ However, when comorbid with neurodegenerative diseases such as Alzheimer’s disease, the efficacy of shunt surgery may be limited.^[9]^

Gerstmann syndrome (GS) is a rare syndrome characterized by 4 cardinal symptoms: acalculia, agraphia, finger agnosia, and left–right disorientation.^[10–13]^ Although incomplete GS, where only some symptoms are present, has been observed,^[14]^ no occurrences of concomitant iNPH and GS have been reported, thus limiting the understanding of the relationship between these 2 conditions.

In this case report, we explored the effects of shunt surgery on a patient diagnosed with iNPH and incomplete GS. This case demonstrates the potential therapeutic avenues for treating atypical neuropsychological symptoms associated with iNPH and provides valuable insights for future treatment strategies.

## 2. Case presentation

### 2.1. Patient history

A 77-year-old woman presented with chief complaints of forgetfulness and confusion in daily activities. The patient’s symptoms had gradually worsened since January 2023, including difficulty recalling the names of objects and an inability to locate the restroom. After admission to a care facility in November, the patient visited a memory clinic in December, where imaging revealed enlarged ventricles, leading to a suspicion of iNPH. Subsequently, the patient was referred to our hospital in January 2024 for further evaluation.

### 2.2. Neurological findings

On neurological examination, the patient’s level of consciousness was normal, with a Glasgow Coma Scale score of 15 (E4V5M6). The Mini-Mental State Examination (MMSE) score was 4 out of 30, while the Nishimura Dementia Scale,^[15]^ a 12-item cognitive test commonly used in Japan, was 25 points, indicating severe cognitive impairment. Acalculia, agraphia, and finger agnosia were confirmed, and left–right disorientation was also present, but only to a mild degree. As all 4 core symptoms of GS were present but left–right disorientation was not severe, we classified this case as “incomplete” GS. We assessed the severity of left–right disorientation based on clinical observation using a bedside left–right discrimination task, where the patient was asked to point to the left and right limbs and objects in space. The patient was able to correctly identify left and right in some instances but made occasional errors.

### 2.3. Imaging findings

**Figure 1.**
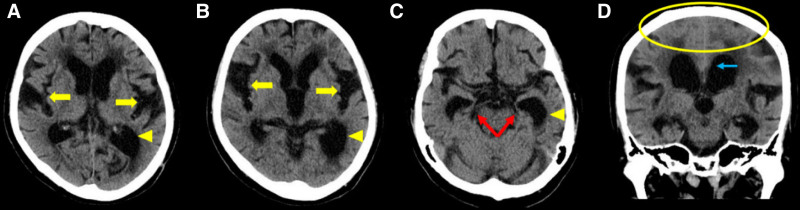
Preoperative computed tomography demonstrating ventricular enlargement with an Evans index of 0.33. (A, B) Horizontal sections showing widening of the Sylvian fissure (yellow arrow) and posterior-dominant enlargement of the left lateral ventricle (yellow arrowhead). (C) A different horizontal section showing the posterior-dominant enlargement of the left lateral ventricle (yellow arrowhead), with marked bilateral hippocampal atrophy (red arrow). (D) Coronal section demonstrating a positive “disproportionately enlarged subarachnoid space hydrocephalus” (DESH) sign (within the yellow box) and a callosal angle of <90° (blue arrow). DESH = disproportionately enlarged subarachnoid space hydrocephalus.

Brain computed tomography scan revealed ventricular enlargement with an Evans index of 0.33, along with widening of the Sylvian fissures, pronounced ventricular dilation toward the left posterior region, and bilateral hippocampal atrophy. In addition, a positive “disproportionately enlarged subarachnoid space hydrocephalus” (DESH) sign and a callosal angle of <90° were observed (Fig. 1).

### 2.4. Treatment course

**Figure 2.**
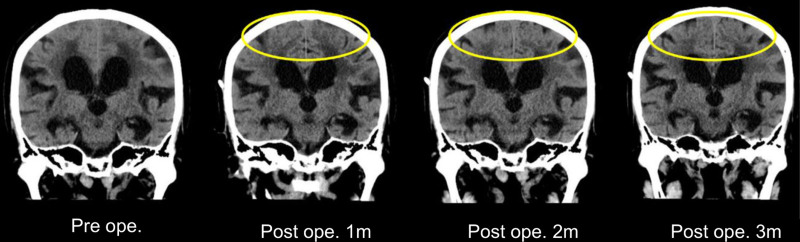
Disproportionately enlarged subarachnoid space hydrocephalus (DESH) sign progression. The narrowing of the high convexity sulci and subarachnoid space, which was observed preoperatively, began to improve 1 mo after surgery, with continued improvement up to 3 mo postoperatively (within the yellow box). However, no significant changes were noted in the widening of the Sylvian fissure. DESH = disproportionately enlarged subarachnoid space hydrocephalus, m = mo.

**Figure 3.**
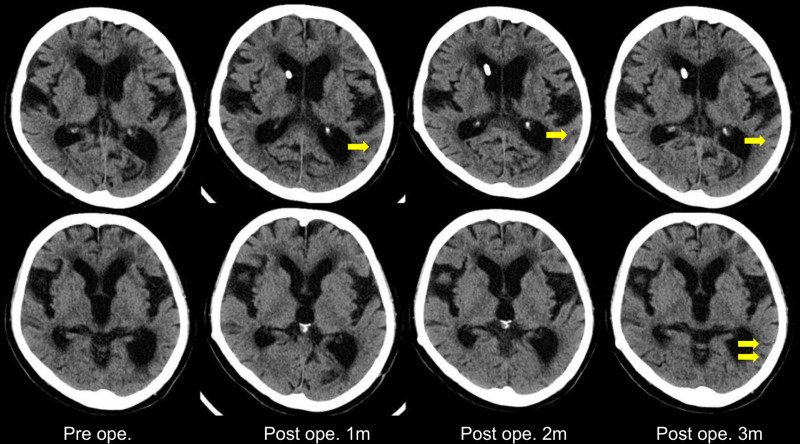
Lateral ventricle progression. The Evans index decreased slightly from 0.33 preoperatively to 0.32 at 3 mo postoperatively. The posterior-dominant enlargement of the lateral ventricles showed minimal improvement, with no major change. However, the sulci in the left temporal lobe became more distinct 3 mo after surgery (yellow arrow). m = mo.

In February 2024, the patient underwent ventriculoperitoneal (VP) shunt surgery using a CODMAN^®^ CERTAS^®^ Plus adjustable pressure valve (Integra Lifesciences, Princeton, NJ). The postoperative course was favorable. One month after surgery, the patient’s MMSE score improved to 9, and by 3 months, it had further improved to 11. Neuropsychologically, left–right disorientation resolved within 1 month postoperatively, accompanied by apparent recovery of agraphia and anomia. Finger agnosia improved after 2 months, while acalculia was partially improved by the third month (Table 1). Imaging studies revealed improvements in the DESH sign 1 month postoperatively and continuing through 3 months (Fig. 2). However, we observed no significant change in the ventricular enlargement, with the Evans index shifting from 0.33 preoperatively to 0.32 at 3 months postoperatively. The posterior predominance of left lateral ventricular dilation and Sylvian fissure widening also showed minimal change. Nevertheless, the sulci in the left temporoparietal lobe became more pronounced 3 months after surgery (Fig. 3).

**Table 1:** Progression of the 4 cardinal symptoms of Gerstmann syndrome.

Symptom	Preoperative	Postoperative. 1 M	Postoperative 2 M	Postoperative 3 M
Left–right orientation	△	○	○	○
Calculation	✕	✕	✕	△
Finger agnosia	✕	○	○	○
Writing ability	✕	△	○	○

○ = normal, △ = impaired (some function remains but is noticeably affected), ✕ = deficient (near-total loss of function), M = mo.

## 3. Discussion

In the present case, we observed significant improvements in the patient’s MMSE score and incomplete GS symptoms, including accompanying anomia, following VP shunt surgery. Similar early cognitive gains after shunting have been reported in larger series, with about half of patients showing meaningful improvements within 3 months.^[16,17]^ The MMSE score showed a rapid improvement 1 month postoperatively, which coincided with the improvement in the DESH sign, suggesting that the redistribution of cerebrospinal fluid (CSF) following the shunt surgery contributed to a temporary recovery in cognitive function. However, the stagnation in improvement after 2 months postoperatively may be related to the bilateral hippocampal atrophy observed on imaging, indicating a possible coexistence of Alzheimer’s disease.^[18]^ Pathological findings consistent with Alzheimer’s disease exist in 31% to 50% of patients with iNPH, and this comorbidity can limit cognitive recovery following shunt surgery.^[19–21]^ When Alzheimer’s disease is suspected, the cognitive improvement following shunt surgery is often limited. Therefore, introducing more precise diagnostic methods, such as amyloid positron emission tomography (PET) or CSF testing, is recommended in such cases.^[22]^

GS is often accompanied by other neurological symptoms beyond the 4 cardinal signs.^[23–25]^ In particular, various types of aphasia frequently accompany GS,^[14,26,27]^ and anomia was present in the present case.^[24]^ The improvement of both the symptoms of incomplete GS and anomia postoperatively may relate to the resolution of the narrowing of the sulci in the left temporoparietal lobe.

The mechanism of GS is related to damage to specific cortical regions or disconnection between functionally related areas.^[12,23,25]^ These findings support the view that disruption of long-range parieto-frontal connections – rather than circumscribed cortical necrosis – underlies the tetrad of GS. Functional mapping and callosal disconnection studies in epilepsy patients have further reinforced this concept.^[28]^ Functional mapping studies in patients with epilepsy have confirmed that the areas responsible for each of the 4 cardinal symptoms are located in different parts of the dominant parietal lobe cortex, with the lower region of the superior parietal lobule being a common area of involvement.^[23]^ For example, acalculia has been associated with the superior parietal lobule and the supramarginal gyrus, whereas agraphia is linked to the superior parietal lobule. This anatomical convergence corresponds to the “Gerstmann core” centered on the left intraparietal sulcus that was recently delineated by a meta-analytic connectivity study.^[29]^ Left–right disorientation involves the upper part of the angular gyrus, while finger agnosia is related to both the angular gyrus and the lower part of the superior parietal lobule. In the present case, preoperative imaging showed left-posterior-dominant ventricular enlargement, which likely caused compression of the superior parietal lobule and angular gyrus. The shunt surgery may have relieved this compression, resulting in the recovery of associated neuropsychological functions.

### 3.1. Limitations

This study had some limitations. First, it was a single case report, and as such, the findings may not be generalizable to all patients with iNPH and incomplete GS. Second, long-term follow-up data were not yet available, and continued monitoring is necessary to assess the sustained effects of shunt surgery on neuropsychological function. Third, although imaging findings suggested possible coexisting Alzheimer’s disease, we did not perform additional diagnostic evaluations such as amyloid PET or CSF analysis, leaving some uncertainty regarding the underlying pathology of the patient. Future studies with a larger cohort and comprehensive diagnostic assessments are warranted to further clarify the relationship between iNPH, GS, and neurodegenerative conditions.

## 4. Conclusions

In the present case, we observed significant improvements in cognitive function and neuropsychological symptoms following VP shunt surgery in a patient with iNPH and incomplete GS. iNPH is recognized as a reversible form of dementia, and the improvement in MMSE scores, as well as the resolution of left–right disorientation, anomia, and finger agnosia following shunt surgery, strongly suggests the efficacy of this surgical intervention for iNPH. However, we also observed bilateral hippocampal atrophy in the present case, raising suspicion of coexisting Alzheimer’s disease. Despite this, the marked short-term improvement following surgery indicated that even when Alzheimer’s disease is suspected, timely intervention may provide meaningful improvements in cognitive function and quality of life.

Early diagnosis of comorbid neurodegenerative diseases is essential for predicting the limits of treatment efficacy; however, the present case demonstrated that shunt surgery can still produce positive outcomes in hydrocephalus cases. Thus, although additional diagnostic tools such as amyloid PET and CSF analysis are recommended in cases where neurodegenerative diseases are suspected, emphasizing that shunt surgery can yield symptomatic improvements even in patients with concurrent conditions is equally important. Continuous long-term follow-up and regular neuropsychological evaluations are necessary for developing more individualized treatment plans.

## Acknowledgments

We express our sincere gratitude to the staff of Fukujukai Adachi Tobu Hospital for their support in patient care and data collection. In particular, we thank Head Nurse Tsubasa Ide for her dedicated care and valuable contributions throughout the treatment process of the patient.

## Author contributions

**Data curation:** Kosei Goto, Atsuya Suzuki.

**Investigation:** Takuto Nishihara.

**Project administration:** Nobuo Kutsuna.

**Supervision:** Nobuo Kutsuna.

**Visualization:** Kotaro Makita.

**Writing – original draft:** Kosei Goto.

**Writing – review & editing:** Nobuo Kutsuna, Kotaro Makita.
